# *In Vivo* Assessment of Phage and Linezolid Based Implant Coatings for Treatment of Methicillin Resistant *S*. *aureus* (MRSA) Mediated Orthopaedic Device Related Infections

**DOI:** 10.1371/journal.pone.0157626

**Published:** 2016-06-22

**Authors:** Sandeep Kaur, Kusum Harjai, Sanjay Chhibber

**Affiliations:** Department of Microbiology, Panjab University, Chandigarh-160014, India; University Hospital Münster, GERMANY

## Abstract

*Staphylococcus* comprises up to two-thirds of all pathogens in orthopaedic implant infections with two species respectively *Staphylococcus aureus* and *Staphylococcus epidermidis*, being the predominate etiological agents isolated. Further, with the emergence of methicillin-resistant *S*. *aureus* (MRSA), treatment of *S*. *aureus* implant infections has become more difficult, thus representing a devastating complication. Use of local delivery system consisting of *S*.*aureus* specific phage along with linezolid (incorporated in biopolymer) allowing gradual release of the two agents at the implant site represents a new, still unexplored treatment option (against orthopaedic implant infections) that has been studied in an animal model of prosthetic joint infection. Naked wire, hydroxypropyl methylcellulose (HPMC) coated wire and phage and /or linezolid coated K-wire were surgically implanted into the intra-medullary canal of mouse femur bone of respective groups followed by inoculation of *S*.*aureus* ATCC 43300(MRSA). Mice implanted with K-wire coated with both the agents i.e phage as well as linezolid (dual coated wires) showed maximum reduction in bacterial adherence, associated inflammation of the joint as well as faster resumption of locomotion and motor function of the limb. Also, all the coating treatments showed no emergence of resistant mutants. Use of dual coated implants incorporating lytic phage (capable of self-multiplication) as well as linezolid presents an attractive and aggressive early approach in preventing as well as treating implant associated infections caused by methicillin resistant *S*. *aureus* strains as assessed in a murine model of experimental joint infection.

## Introduction

Staphylococcus is a major pathogen involved in post arthroplasty and orthopaedic implant related infections [1–3]. Coagulase-negative staphylococci (CoNS) account for 30–41% of such cases. *S*.*aureus*is second in line, being involved in 12–39% of cases reported [4–6]. The increasing prevalence of methicillin-resistant *Staphylococcus aureus* (MRSA) represents a significant healthcare burden [7–9]. In orthopaedic implant infections, *S*. *aureus* is more virulent than CoNS and if infected with a MRSA strains, the patient has the worst outcome with more post-infection sequelae than if infected with a sensitive *S*.*aureus* strain [10]. One potential therapeutic strategy is local drug delivery where antibiotics delivered locally at the implant site in high concentration can take care of pathogenic bacteria. This can be achieved either by using an adequate carrier or by coating the implants (stainless steel or titanium implants) with polymers loaded with antimicrobial agent [11–13]. Large number of delivery strategies have been used till date. One of the oldest in use are bone cements [i.e Poly(methyl methacrylate (PMMA)]that are loaded with antibiotics [14,15]. However, the major drawback of such system is that PMMA used is not biodegradable and is itself prone to microbial adhesion and biofilm formation [16–19]. Also, such systems allow long term slow release of antibiotic, exposing bacteria to sub-MIC concentrations that enhance emergence of resistant mutants and infection relapse [20–23]. In addition, one of the major drawbacks of antibiotic based delivery systems is the local tissue toxicity towards osteoblast activity (hindering with the process of bone healing) exhibited by most of the antibiotics used [24–27]. Silver coatings although represent an attractive antimicrobial strategy for local delivery but issues of silver toxicity and emergence of bacterial resistance to silver needs to be addressed [20,28,29]. Hence, there is a need for developing newer and safer agents for local delivery at implant site. Efficacy of local delivery system employing lytic phage and linezolid impregnated in a biodegradable polymer coated on K-wires (K-wire is commonly used orthopaedic implant for pin fixation and anchoring of skeletal traction) has already been studied *in vitro* [30]. Phages showed complete biocompatibility and stability with HPMC with steady release till 96 h from coated K-wires. The dual delivery system was able to significantly decrease the *in vitro* bacterial adhesion and colonisation on the implant as compared to naked wire. Also, dual coating involving combination of two antimicrobial agents significantly reduced (<10^−9^) the frequency of emergence of resistant mutants *in vitro* [30, 31].

This delivery system offers the advantage of using broad spectrum lytic phage (active against resistant and sensitive *S*.*aureus* strains) which has the ability to self-replicate (auto dosing) without any issues of adverse effect or local tissue toxicity [30, 32–34]. The second component is linezolid, a bacteriostatic agent that works by inhibiting the formation of initiation complex during bacterial protein synthesis. Its effectiveness against Gram-positive cocci (streptococci, enterococci, staphylococci), 100% bioavailability allowing easy intravenous to oral switching without dose adjustments [35,36], good bone and tissue penetration reaching high concentrations in musculoskeletal tissues (skin, synovial fluid and) and effectiveness against drug resistant isolates [37–40] favours its use against prosthetic joint infections. Although there are few reports that focus on the local elution of linezolid from acrylic bone cement spacers and polymethylmethacrylate (PMMA) beads [40–42] but, in vivo profile of use of such linezolid-loaded polymers in experimental animals has not been looked into.

## Results

### Establishment of *S*.*aureus* mediated murine model of joint infection

**Fig 1:**
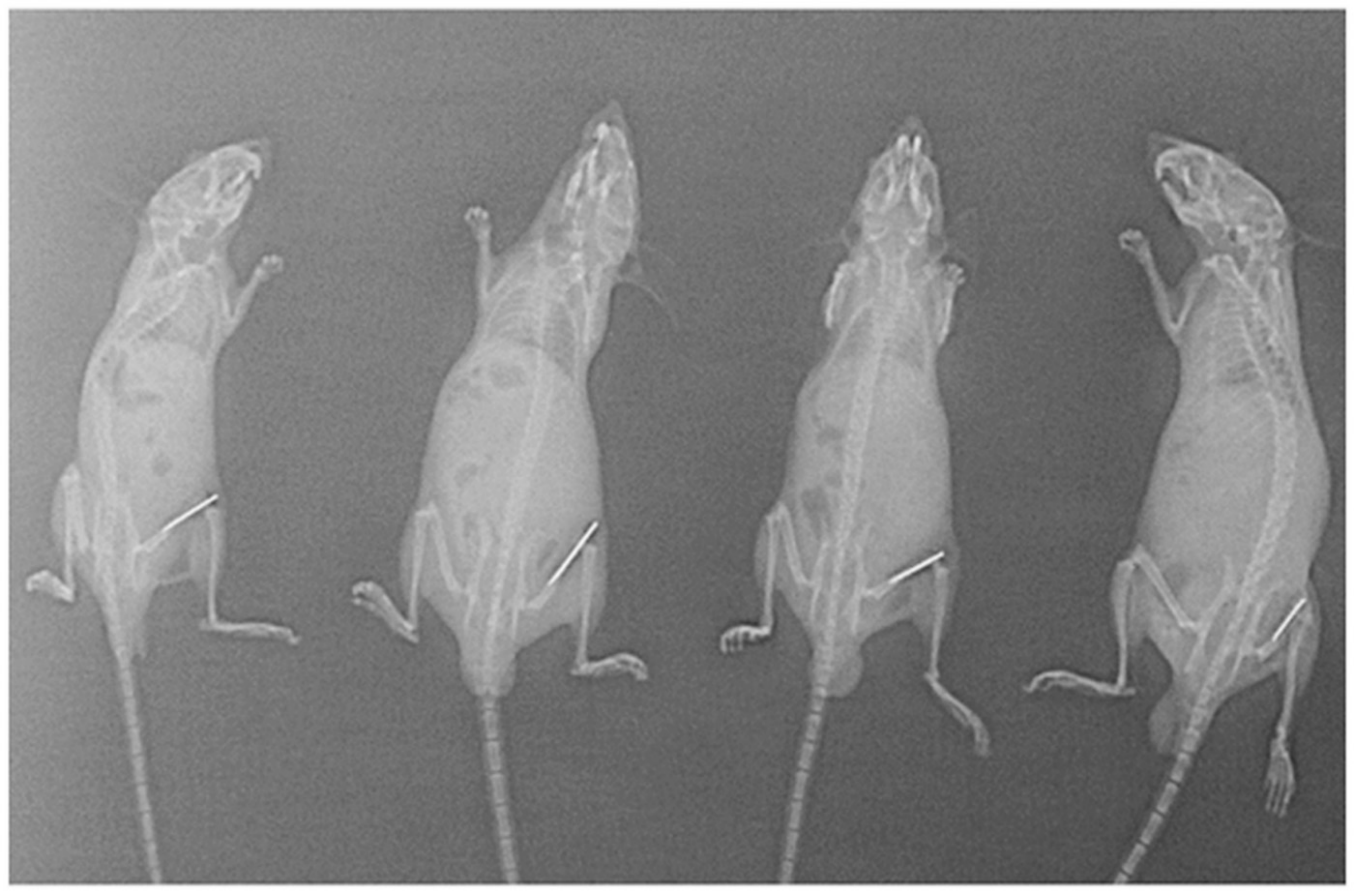
Radiographs of mice implanted with K-wire showing the correctness of implanted wire after the completion of surgery.

To develop model of post-arthroplasty infection, an orthopaedic-grade, K-wire was surgically placed into the femur followed by inoculation of *S*.*aureus* into the joint space before suturing. Correct placement of the wire in each animal was confirmed by radiography (X-ray) as shown in Fig 1. Only those animals with correctly placed K-wire were selected for experimentation. However, all the animals that were surgically operated had correctly placed wire. Since the aim of this study was to assess the efficacy of phage coated and/or antibiotic coated K-wires on the course of joint infection, therefore doses higher than 10^7^ and10^8^ were not selected as these led to development of a severe purulent joint infection with > 50% mortality (observed within 48–72 h of inoculation) in mice respectively (S1 File, S1 Table, S1 Fig). In order to develop a persistent joint infection associated with marked inflammation, pain, increased swelling, decrease in mobility/locomotion, stiffness in joint but with no death, a lower dose of 10^6^ CFU/ml was chosen as the infectious dose. This dose helped us to follow the infection over a period of 20 days.

### Oedema Scoring

A constant increase in oedema of the affected joint was observed (Table 1) following implantation of naked wire into the intramedullary canal of mouse femur bone. The oedema was maximum on day 3 and 5 and mild oedema persisted till day 15. Similarly the animals with polymer coated implants showed consistent increase in oedema of the affected limb which declined by day 15. However, in case of mice implanted with phage coated (H-P) and linezolid coated wire (H-L), moderate oedema was seen on day 1 and it continued on the following days. No visible oedema was seen day 10 onwards. In case of mice with dual coated implants (i.e group 5), only mild oedema was observed in the affected limb which resolved by day 10.

**Table 1:** Oedema and lesion score of mice following infection with *S*.*aureus* 43300.

Days	Naked wire(Gr.1)	HPMC coated wire (Gr.2)	Phage coated wire (Gr.3)	LNZ coated wire (Gr.4)	Dual coated wire (Gr.5)
Day 1	2	2	2	2	1
Day 3	3	3	2	1	1
Day 5	3	3	1	1	1
Day 7	2	2	1	1	1
Day 10	2	2	0	0	0
Day 15	1	0	0	0	0

### Functional Healing

#### Locomotor activity

**Fig 2:**
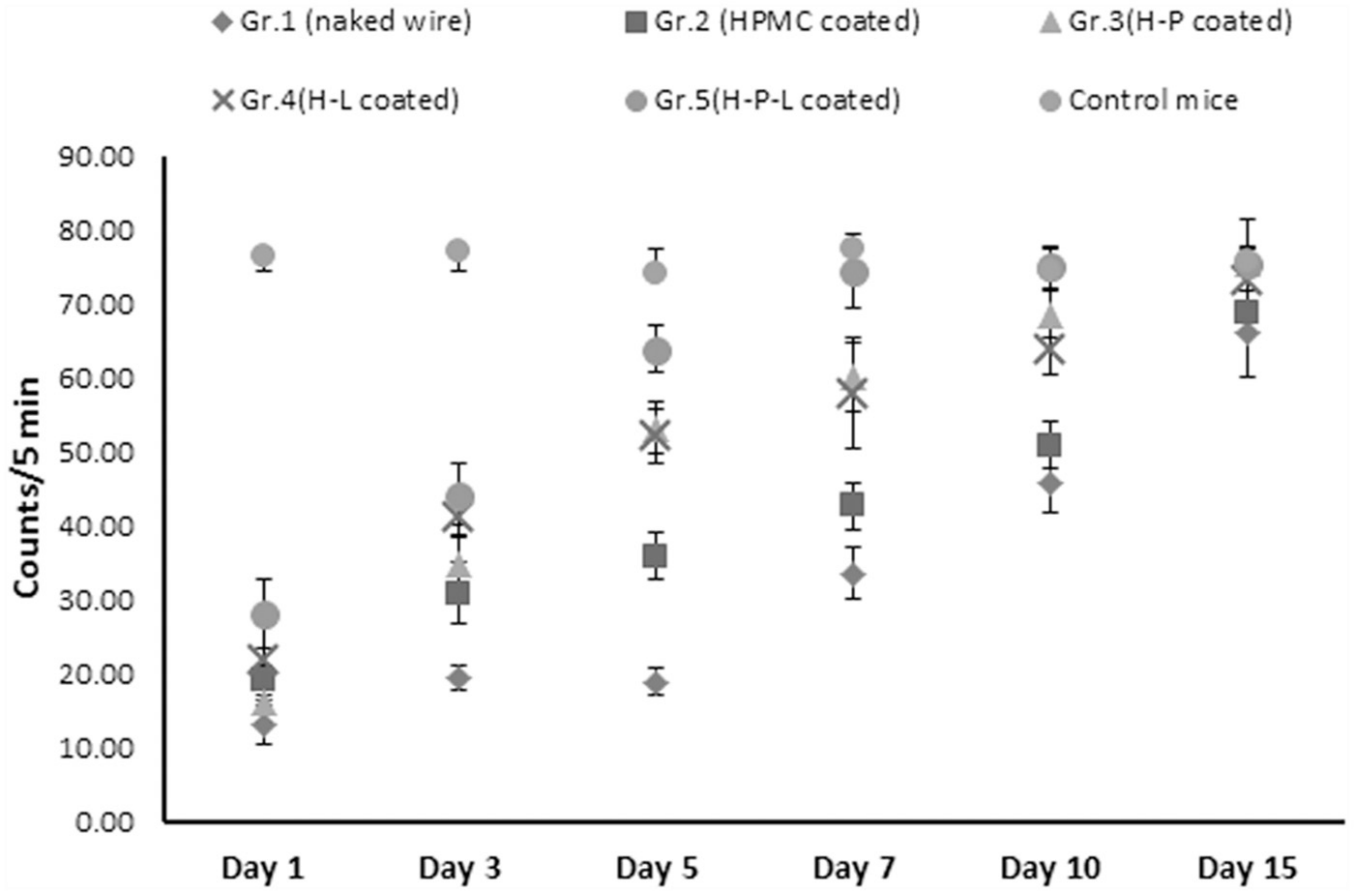
Mean locomotor activity of mice (n = 6) recorded as counts/5 min on different days post surgery in comparison to normal age matched mice (n = 6). Error bars represent S.D.

Both ambulation and rearing was recorded (Fig 2) for each mouse by acto-photometer and expressed as total counts per 5 min per mouse. Healthy mice (age matched) were included as controls to determine the normal locomotor activity, with an average activity of 75.8 counts/5 min. Animals with either naked or HPMC coated wires (group 1 and group 2) showed significantly decreased mobility initially. Even by day 10, mice showed only 60% locomotor activity as compared to healthy mice.

Mice with phage coated implants (H-P) showed low locomotion on day 1 and 3 but showed significant improvement in locomotion by day 5 (~70% locomotion activity) and resumed normal locomotor activity by day 10. Similarly, mice with linezolid coated implant (H-L) resumed 90% locomotion by day 10. Significant improvement in locomotion was seen in mice implanted with dual coated wire (H-P-L) They resumed 84% locomotor activity by day 5 itself and resumed normal activity thereafter.

#### Rotarod test

Motor function evaluation was done by rota rod test and fall off time was recorded for each mouse stay. Time of more than 180 seconds (>3 min) on the rota rod is the normal value for mice. Animals fitted with naked wire (Table 2) showed fall off time of 39 sec even by day 10. By day 15, rodents were able to prolong this time to 111sec. In case of mice implanted with phage coated (H-P) and linezolid coated wire (H-L), fall off time of 40 sec was recorded on day 3 and increased to >180 seconds by day 7. However, mice with dual coated wires (H-P-L) showed faster improvement in their balancing potential with fall off time of 131 sec by day 5 itself followed by resumption of>180 sec thereafter.

**Table 2:** Mean fall off time (second) of mice (n = 6), surgically implanted with K-wires on the basis of rota rod motor function test.

Days	Naked wire(Gr.1)	HPMC coated wire (Gr.2)	Phage coated wire (Gr.3)	LNZ coated wire (Gr.4)	Dual coated wire (Gr.5)
Day 1	1.96±0.52	3.83± 1.24	15.63±2.19	16.15±3.24	16.61±1.87
Day 3	8.37±2.47	11.10± 2.07	42.27±4.30	41.36±4.88	56.31±2.99
Day 5	18.16±2.72	21.55±3.80	114.67±6.54	104.36±3.80	120.00±6.21
Day 7	20.61±1.59	32.00±4.69	>180	>180	>180
Day 10	39.37±3.63	52.96±3.68	>180	>180	>180
Day 15	113.40±4.84	>120	>180	>180	>180

(>180 seconds: normal value for rotarod test). Each value represents mean ± S.D of n = 6 mice per time point.

### Bacterial burden

**Fig 3:**
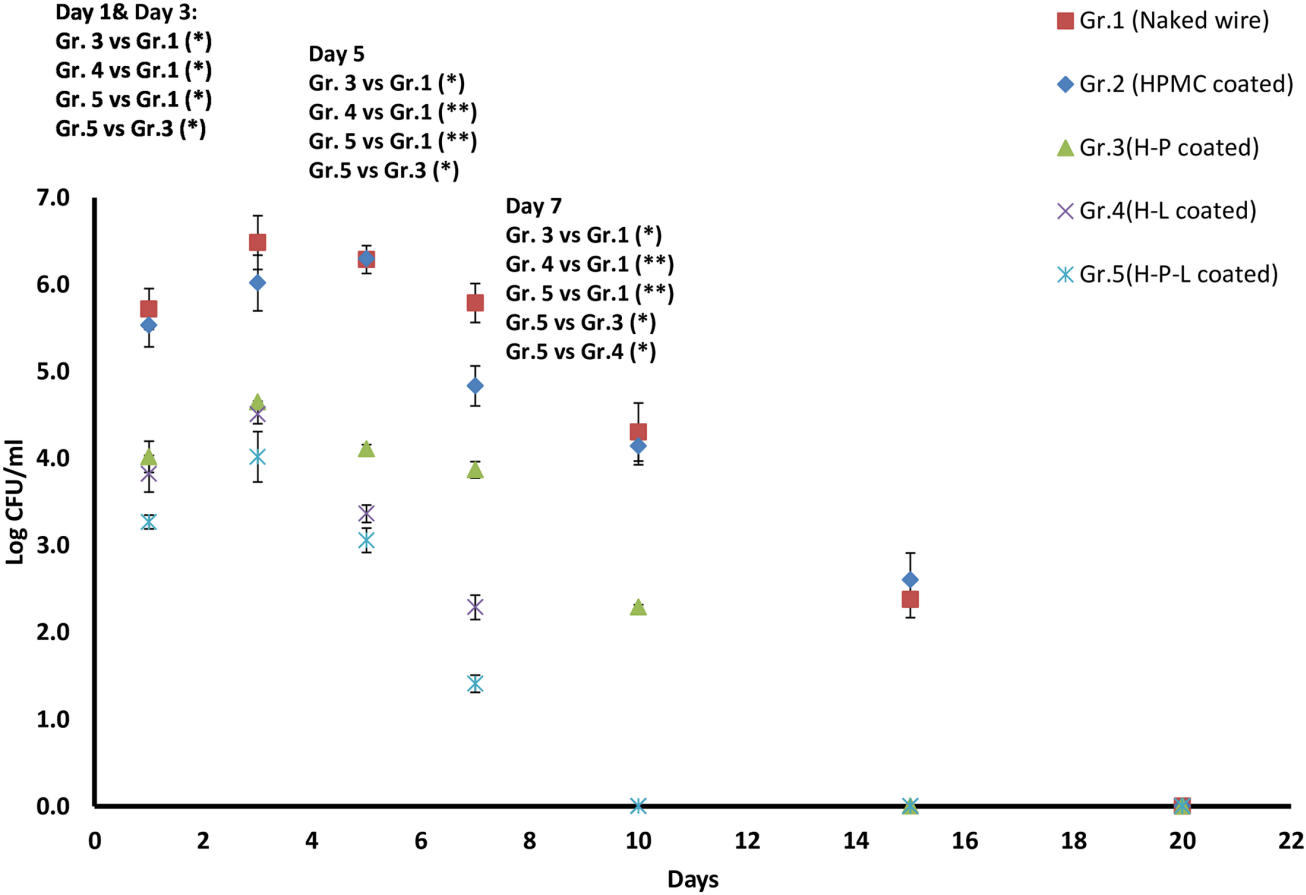
Bacterial load (Log CFU/ml) on implanted K-wire in mice on different days post infection with *S*. *aureus* 43300. Data points represent mean ± S.D of three independent values. p values among groups have been determined where (*) represent p<0.05 and (**) represent p<0.01.

The bacterial burden on the implanted wire was determined (Fig 3). Time dependent increase in bacterial burden on implanted wire was observed with peak bacterial load of ~6 logs adhered on naked (group1) as well as HPMC coated wires (group 2) by day 3. Consistent bacterial load of 2 logs was detectable even on day 15. However, all treatment groups showed significant decrease (p <0.05) in the number of adhered bacteria in comparison to group 1(naked wire) and group 2 (HPMC control) animals. Mice implanted with phage coated wire (H-P) showed a consistent load of ~4 logs until day 5 that gradually declined by day 7 onwards. Animals of both the groups i.e those implanted with linezolid coated wire (H-L) as well as dual coated wire (H-P-L) showed significantly reduced adherence of bacteria on K-wires on all days with highly significant reduction of ~3 log by day 5 (p <0.01) as compared to infection control group. Minimum adherence was seen in mice implanted with dual coated wire (group 5) on all days.

**Fig 4:**
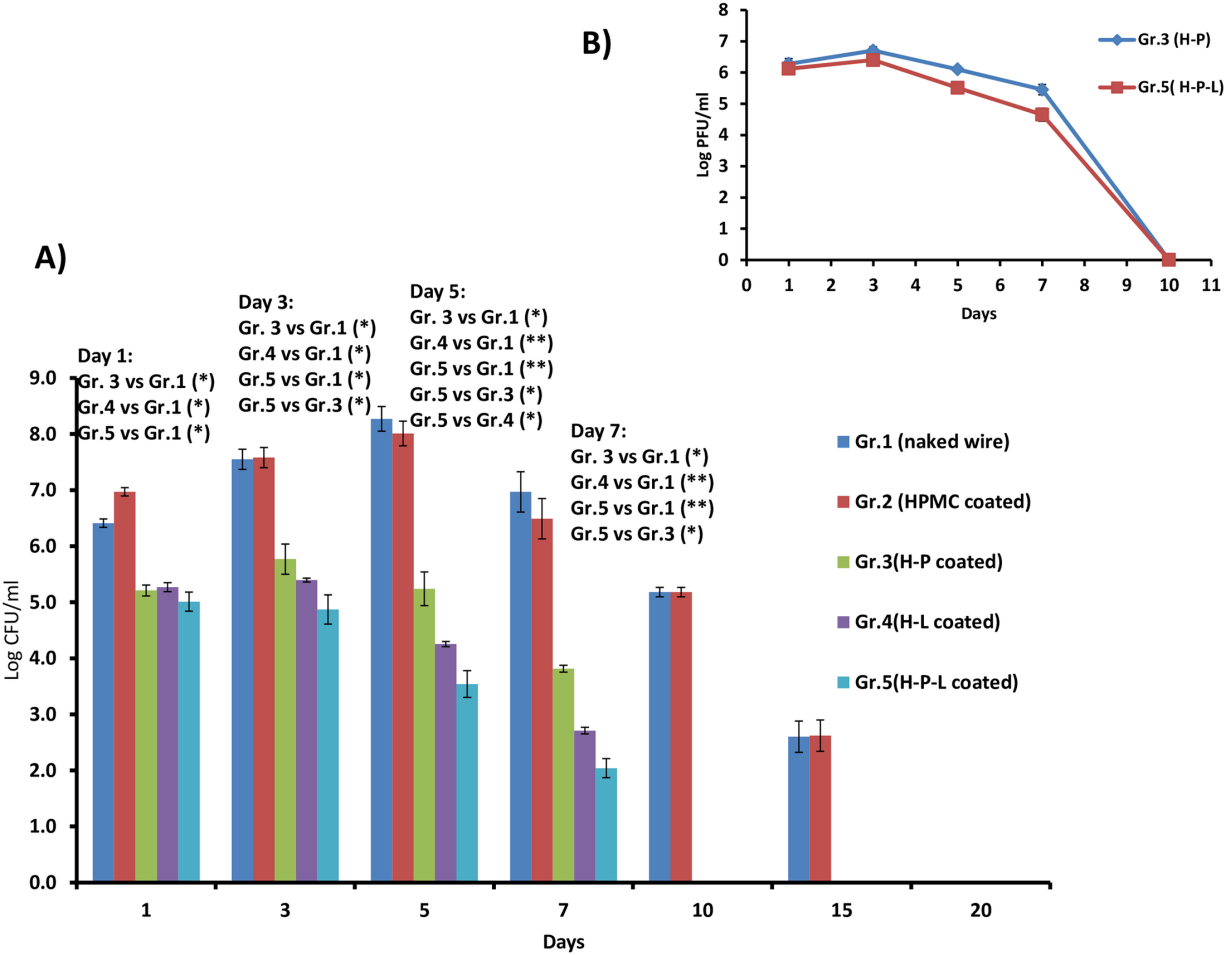
A) Bacterial load (Log CFU/ml) in the adjoining joint tissue of mice on different days post infection with *S*. *aureus* 43300 B) Phage titer (Log PFU/ml) in the adjoining joint tissue of mice on different days post infection with *S*. *aureus* 43300. Each data point represents mean ± S.D of three (n = 3) values at each time point. p values among groups have been determined where (*) represent p<0.05 and (**) represent p<0.01.

The bacterial burden in the surrounding joint tissue was also determined. As shown in Fig 4, the bacterial load in joint tissue (surrounding the infected implant) of mice implanted with naked wire (group 1) as well as HPMC coated wire (group 2) showed consistent increase from ~6 logs on day 1 and reaching ~8 logs by day 5. Phage coated mice (H-P) showed a peak on day 3 followed by significant reduction (p<0.05) of >3 logs on day 5 and day 7 as compared to group 1 and group 2. Similarly, linezolid coated groups (H-L) also showed significant decrease of ~4 logs w.r.t mice of group1 by day 5 (p<0.01) and sterile tissue was obtained by day 10. Group 5 mice coated with dual implants (H-P-L) showed maximum decrease among all the treatment groups. Peak load of 5.01±0.035 log CFU/ml was detected on day 1 followed by significant decrease (p<0.05) by day 3 itself. Highly significant decrease of ~ 4.5 logs (p <0.01) was obtained on day 5 and day 7 as compared to group 1 (naked wire) and group 2 (HPMC coated). Joint tissue was sterile with no load detected after day 7.

Phage released in the surrounding joint tissue from coated K-wires was also determined in the tissue homogenates of mice fitted with phage coated (group 3) and dual coated wire (group 5). The results depicted in Fig 4 show that phage titer in both the groups reached ~6 log cycles on day 1. The titer showed a slight increase to 6.71 log PFU/ml (group 3) and 6.42 log PFU/ml (group 5) by day 3 respectively. This was followed by decrease on day 5 in both the groups with titer of 5.45 and 4.65 log PFU/ml achieved by day 7. No plaque were detected thereafter.

### Tissue PCT levels

**Fig 5:**
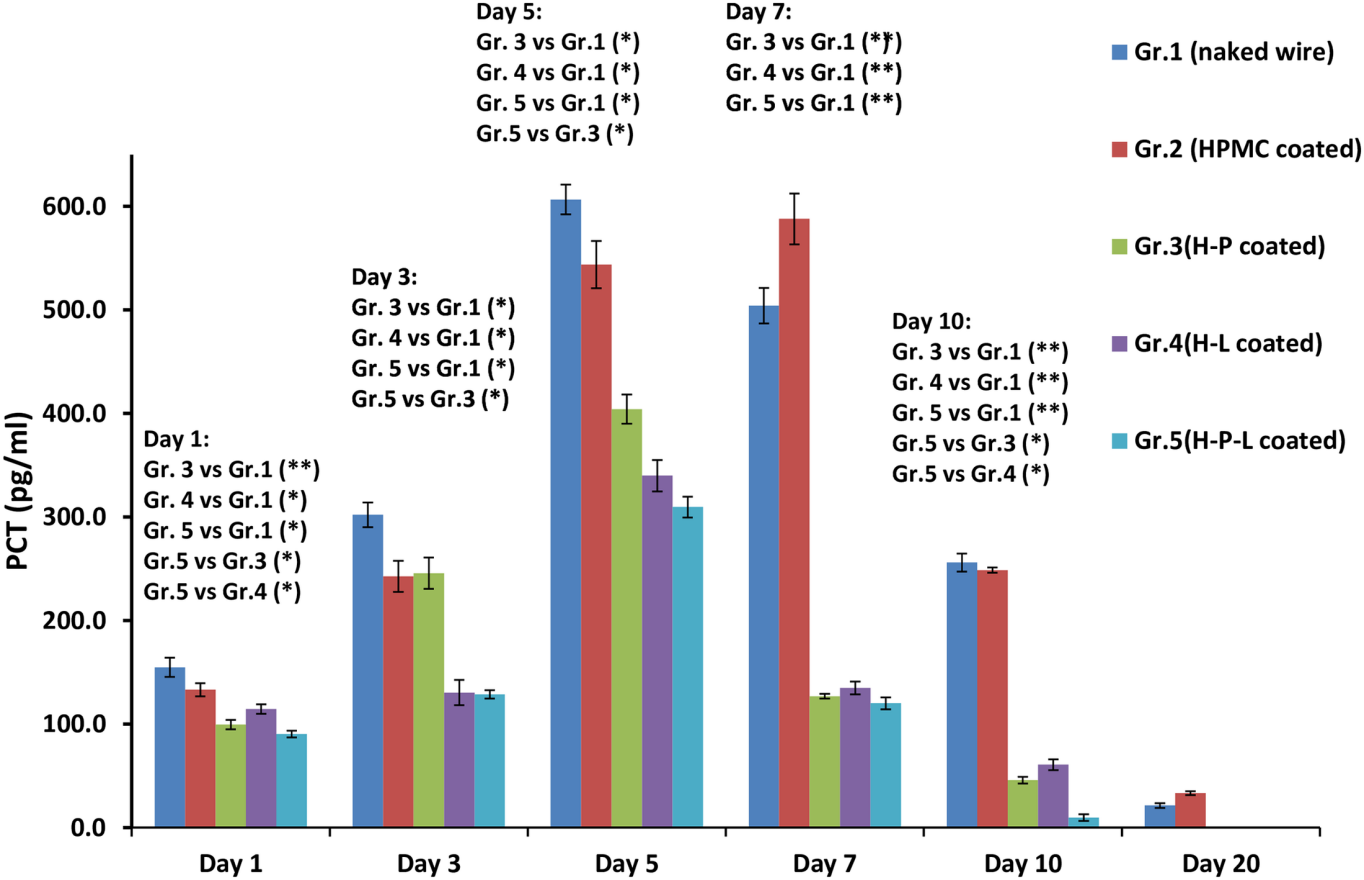
Pro-calcitonin (PCT) levels in joint tissue of mice on different days post infection with *S*.*aureus* 43300. Each data point represent mean ± S.D of three values. p values among groups have been determined where (*) represent p<0.05 and (**) represent p<0.01.

As depicted in Fig 5, animals of group1 (implanted with naked wire) showed rising PCT levels in tissue samples on subsequent days with the progression of infection. Peak concentration of ~600 pg/ml was detected on day 5 and the levels were still high on day 7 as well (504 pg/ml). PCT levels significantly dropped to <30 pg/ml after day 10. Similarly, animals of group 2 (HPMC control group) also showed a time dependent increase in tissue PCT levels reaching high concentrations of 543.7 and 588 pg/ml by day 5 and 7 respectively. All treatment groups showed comparatively lower levels of PCT at all time points. Mice implanted with phage coated (H-P) and linezolid coated wire (H-L) showed rising PCT levels (starting with ~100 pg/ml on day 1) till day 5. Peak was observed on day 5 in phage coated and linezolid coated groups (not exceeding beyond 400 pg/ml). The concentration significantly dropped by day 7 (p<0.05) in both the groups and negligible levels were detected by day 10. The animals with wires having dual coating(H-P-L) showed minimum concentration of PCT as compared to other groups. Peak PCT levels was seen on day 5 (309.6 pg/ml) and by day 7 itself, the levels were significantly lower (p<0.01) as compared to PCT levels detected in naked wire group with an average value of 120 pg/ml only. By day 10 it was beyond the detection limit (<10 pg/ml).

### Cytokine levels

**Fig 6:**
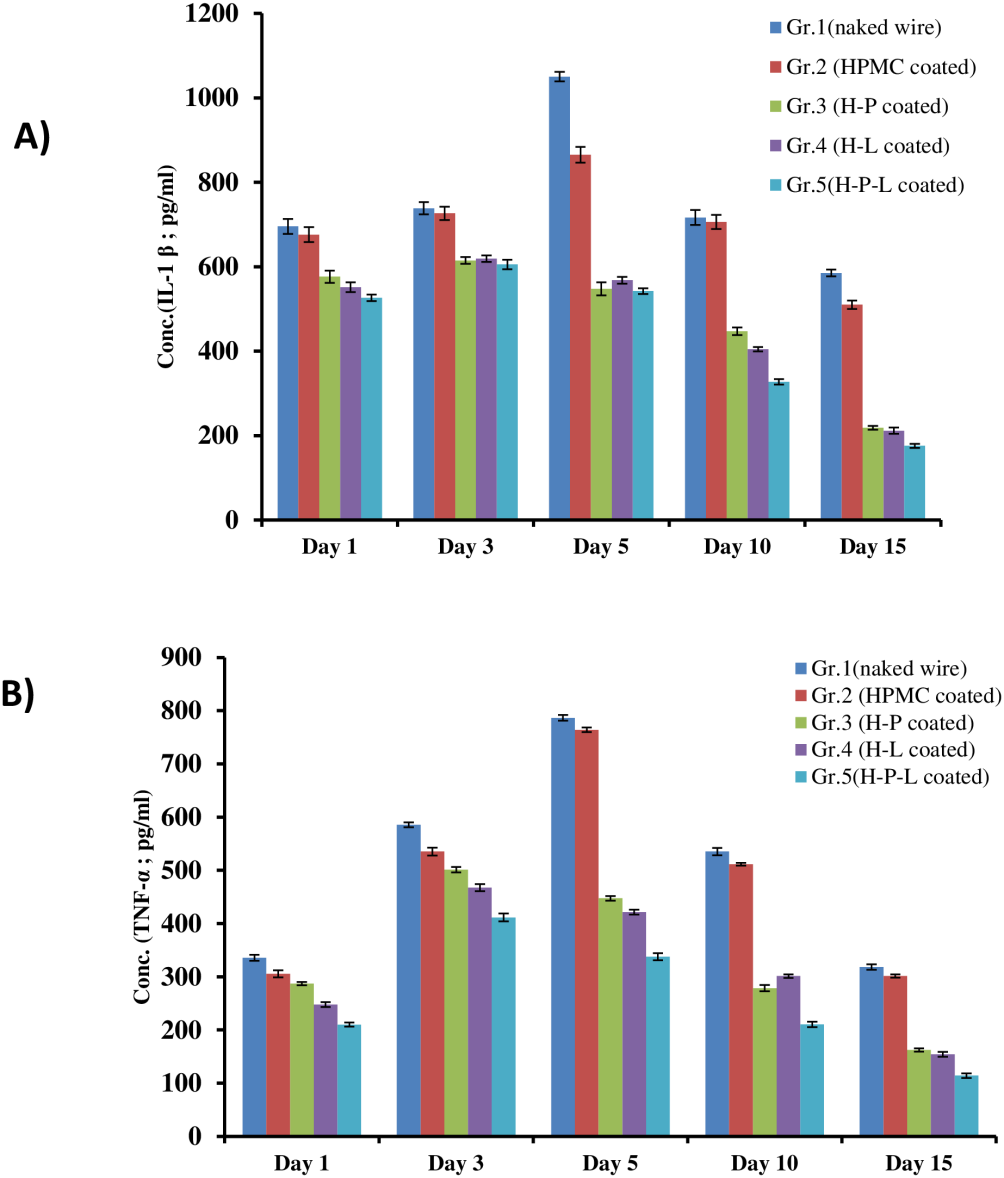
Cytokine levels of A) IL-1β and B) TNF-α in tissue homogenate of mice on different days post infection with *S*.*aureus* 43300. Each data point represent mean ± S.D of three values. Error bars represent S.D.

Levels of cytokine IL-1β and TNF-α in tissue homogenates was determined on different days post infection (Fig 6A and 6B). Animals of group 1 (naked wire) and group 2 (polymer coated) showed constant increase in the levels of IL-1β and TNF-α with peak level obtained on day 5 in both the groups. Cytokine level was significantly less (p<0.05) in all the treated groups as compared to group 1 and 2. Animals of group 3 (phage coated) and group 4 (linezolid coated) showed significantly low levels of this cytokines on all days as compared to untreated mice (p<0.05). However, mice implanted with dual coated wire (group 5) showed minimum levels of IL-1β and TNF-α at all time points. The levels were significantly less than those seen in mice fitted with naked wire (group 1) and HPMC coated wire i.e group 2 (p<0.05). Peak concentrations of ~600 pg/ml and 411 pg/ml of IL-1β and TNF-α respectively were obtained on day 3 and minimal levels were obtained on following days in group 5 (dual coated) mice.

### Histopathology

**Fig 7:**
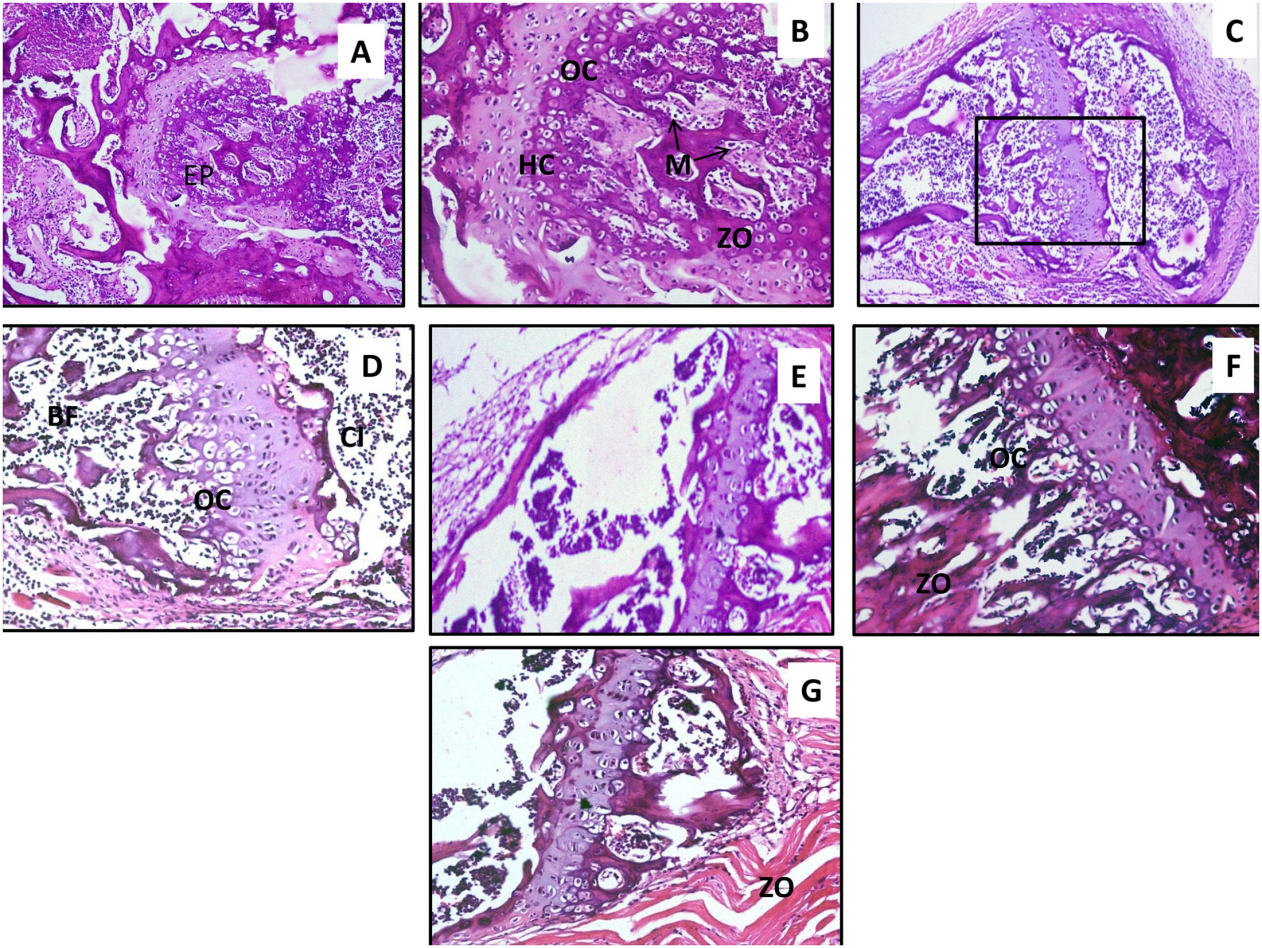
Photo micrograph of A) and B) healthy knee joint tissue of mice (H and E 100X and 200X) C) *S*. *aureus* infected post-operative knee joint tissue of mice implanted with naked wire (gr.1) on day 7 post infection (H and E 100 X) and D) Magnified view of the area (denoted in the rectangular panel) showing infected knee joint with heavy infiltration (H and E 200X). E) Photo micrograph of *S*. *aureus* infected post-operative knee joint tissue of mice implanted with phage coated wire (H-P) on day 7 post infection (H and E 100X) F) Photo micrograph of *S*. *aureus* infected post-operative knee joint tissue of mice implanted with linezolid coated wire (H-L) on day 7 post infection (H and E 200X) G) Photo micrograph of *S*. *aureus* infected post-operative knee joint tissue of mice implanted with dual coated wire (H-P-L) on day 7 post infection (H and E 100X). (HC: Hyaline cartilage; ZO: zone of ossification; EP: epiphyseal growth plate; OC/OB: osteocytes/osteoblasts; BF: bone fragmentation; CI: cellular infiltration).

The extent of tissue injury in the infected knee joint tissue surrounding the implanted wire (placed in the intramedullary canal of mice femur bone) in different groups post treatment was analysed on day 7 post infection. Histology of uninfected healthy knee joint tissue (Fig 7A and 7B) showed an intact epiphyseal growth plate (EP) which acts as site for bone elongation and a rich deposit of hyaline cartilage (HC). Also, clear zone of ossification (ZO) with numerous osteocytes (OC) and osteoblasts was seen adjacent to the growth plate as well as surrounding the marrow (M) extending through the medullary canal. As shown in Fig 7C and 7D, tissue from infection control showed extensive acute and chronic inflammation with infiltration of polymorphonuclear leukocytes and abscesses formation in the joint tissue. Extensive bone fragmentation (BF), new bone formation, and sequester formation was evident. Acute inflammation with foci of polymorphoneutrophils and occasional micro-abscesses was also seen in knee joint tissue of mice implanted with phage coated (H-P) and linezolid coated wire (H-L). However, the degree of infiltration of lymphocytes and plasma cells was comparatively limited as compared to animals with naked wire. Similarly, the histopathology of joint specimens from mice with dual coated implants (Fig 7G) showed lesser degree of cellular infiltration of joint and adjoining soft tissue. Intact epiphyseal growth plate with zone of ossification surrounded by osteoblasts/osteocytes and lesser degree of bone fragmentation was seen. Mild neutrophil infiltration beneath the cartilage tissue was observed, correlating with the healing process.

### Resistant mutants

Bacterial growth detached from implants coated with H-L and H-P-L did not show any colonies appearing on plates supplemented with linezolid (8 mg/L). With broth micro-dilution assay, MIC value of 2 mg/L for linezolid was obtained against the bacteria detached from such implants. No increase in MIC values was seen with bacterial growth obtained from either H-L or H-P-L implants. Similarly, on plaque assay, no colonies were emerged (both from H-P or H-P-L implants) when plated with phage added at MOI-10. Al lower MOI-1, the resulting colonies that emerged were again subjected to phage spot assay and all were susceptible.

## Discussion

The present concept of using phage and linezolid as local delivery agents was evaluated in mouse model of post arthroplasty infection established following the method of Bernthal et al.[43]. While establishing the animal model, initial experimentation showed necrosis of the affected limb and death in mice at a high dose of bacteria. In order to compare the treatment efficacy in terms of decrease in bacterial load, functional healing and inflammation with the untreated group, no mortality was desired and therefore, a lower dose of 10^6^ CFU/ml was used as it led to development of a sustained and persistent self resolving infection over a 15–20 days post-operative period. Mouse is not considered as natural host to *S*.*aureus* [44–47] as they have the ability to clear heavy loads of infection due to efficient recruitment and functioning of neutrophils [44,48–51]. Staphylococcal peptidoglycan is also known to promote clearance of experimentally induced infection in mice [45]. To circumvent this problem, researchers adopt methods to manipulate the immune status of the host animal by inducing neutropenia [52–55], use of hog gastric mucin [56–58], tissue trauma or skin injury [59,60] for successful establishment of infection and death at lower doses. This presents a limitation as it does not closely mimic human condition, where *S*.*aureus* infections can generally be caused by small initial inoculums [45]. Therefore, although a bacterial inoculum of 10^6^ CFU/ml was still high but eventually all the test mice were able to self resolve the implant infection process by day 20.

The signs and symptoms of orthopaedic device related infections (ODRI’s) include persistent pain, fever, tenderness with swelling around the affected joint, decreased mobility and loosening of implant [61,62]. None of the past researchers have studied changes in physical activity (i.e locomotion, joint strength, pain alleviation) to track the progression of orthopaedic implant infection and its treatment efficacy. However, a lot of importance has been stressed on these functional parameters for studying disease progression in case of arthritis (osteoarthritis, septic arthritis, rheumatoid arthritis) in mouse models and related intervention therapies [63–65]. Rajasekaran et al.[66] observed maximum decline in locomotor activity during the period when peak joint thickness occurred in the arthritic mice. Similar observation was made by Frommholz and Illges [67] who also showed an indirect correlation of ankle thickness and locomotor activity in arthritic K/BxN mice. Our study also demonstrates similar correlation between oedema scoring of infected limb and locomotion counts. Maximum decline in locomotor activity was observed on initial days i.e day 1 and 3 when oedema score of 3 was observed in untreated mice (naked as well as HPMC coated wire group). Similarly, the lowest locomotor activity recorded on day 1 correlated with peak oedema score in all treated groups of mice. Locomotion resumed to normal counts by day 5 and oedema of affected joint also improved after day 1 with minimal score thereafter.Rotarod test as another functional parameter was also tested in this study. This is a performance based test to evaluate the animals balance, grip strength, endurance and motor-coordination on a rotating rod [68–70]. It has been used since long by researches to assess the motor function in a number of arthritis based animal models [71–73]. But till date no one has used to access its efficacy in implant infections. Clinically, pain is the single most frequent symptom consistently seen in patients with prosthetic joint infections [74–76]. Pain due to inflammation has a significant impact on motor function and coordination [73]. Moreover, inflammatory mediators such as prostaglandin E2 and inflammatory cytokines (TNF-α, Il-6) have been shown to exacerbate the related pain and decrease in mobility or physical activity in patients with rheumatoid arthritis and other joint diseases [77–79].

In our study, mice with naked wire or HPMC coated wire showed poor balancing act and motor-coordination even on day 10. This is supported by an oedema score of 2 seen in this group of animals. With decrease in inflammation and associated pain, mice are likely to exhibit increase in fall out time and better motor co-ordination on the rotating rod. This was observed in all the treatment groups(resumption by day 7) with maximal effect observed in animals fitted with dual coated implants [increase in fall off time of 131 sec by day 5 itself]. These findings stress on the measurement of pain as an additional readout parameter (functional healing), which needs further assessment in the current joint implant model.

The pro-inflammatory cytokine and tissue PCT levels in the naked wire and treatment groups further support our hypothesis that improved functional healing observed in treated mice was due to decrease in inflammation. PCT is a more specific marker of bacterial inflammation than C-reactive protein or white blood count, as its level does not rise in response to viral infection or other non-bacterial inflammation [80–83]. Also, pro-inflammatory cytokines such as TNF-alpha and IL-1 beta that play an important role in joint diseases are central inducers of joint pain and tissue destruction [84–88]. Pain management therapies as well as drugs with anti-inflammatory potential (e.g anti-TNF based therapy) have been shown to significantly improve motor function and balancing on rotarod test in animal models of joint diseases [88–90]. Naked wire or HPMC wire group animals showed peak PCT levels of 600 pg/ml on day 5 and remained high till day 10. Similarly, cytokine levels of IL-1β, TNF-α showed peak concentration on day 5 which remained elevated even by day 10. Peak inflammation (in terms of oedema score, peak PCT levels and cytokine levels) was seen by day 5 and remained high till day 10. This supports our findings of poor fall off time of 39 seconds and poor resumption to locomotion seen in untreated mice even on day 10 post-infection. In mice with phage and linezolid coated wire, peak PCT levels were seen on day 5 but were significantly lower than the untreated mice. Thereafter, PCT levels dropped significantly below the detection limit. Similarly, levels of IL-1β and TNF-α showed a peak concentration on day 3 and 5 with significant reduction thereafter. Locomotion in this group of mice also improved after day 5 with 90% locomotor activity seen by day 10. Also, motor function resumed to 180 sec fall off time by day 7 itself, correlating well with the decrease in inflammatory parameters.

Maximum improvement in functional healing was observed in mice fitted with dual coated wire (phage and linezolid coated). Peak PCT levels reached only 300 pg/ml on day 5 and reduced thereafter. The levels were lowest among all the other test groups. Also, pro-inflammatory cytokine levels showed a peak level of 400 pg/ml on day 3 with minimal levels by day 10. This group of mice showed fastest resumption of locomotor activity [resumed 84% locomotor activity by day 5 itself and improved balancing act (fall out time of 120 second by day 5)] in the shortest time as compared to all other test groups. Histopathological analysis also corroborated these findings as minimum infiltration of inflammatory cells and maximum healing of affected joint tissue was seen in animals having dual coated implant wire.

Increased oedema and increased levels of inflammation observed in untreated mice were clearly due to the MRSA induced infection of the joint tissue. Therefore, the coated implants were tested for their ability in controlling bacterial adherence on the wire as well as bacterial burden in the surrounding joint tissue. Maximum bacterial adherence onto the naked as well as polymer coated wire was seen on day 3 and 5 reaching ~6 log CFU. Mice with phage coated wire although showed maximum adherence of ~4 logs on wire on initial days but by day 5 and beyond, the phage was able to effectively control the multiplying population on the wire with significant reduction by day 7 and beyond. Also, phage released at the implant site was able to control the tissue bacterial burden with significant reductions (>3 logs) seen on day 5 and sterile tissue obtained by day 10. The maximum reduction in bacterial adherence on K-wire as well in the joint tissue from day 1 itself was seen in animals fitted with dual coated wire with sterile tissue obtained by day 10. Phage titer remained high (~6 log PFU/ml) till day 3 with slight reduction on day 5 and this led to complete eradication of the adhered population with minimal load seen on day 7 in dual coated group. No bacterial burden was detected in tissue and on wire in animals fitted with dual coated wire. As a result, no plaques were detected by day 10 as phage survived only as long as its host bacteria was present and later got rapidly cleared from body. However, in case of animals implanted with phage coated wire, minimal phage titer was detected even on day 10 as bacterial load of ~2 log cycles was seen adhered on the implanted wire. This observation clearly indicates that although phage alone was able to contain the infection process and reduce the bacterial load in tissue, but it took a longer time with initial delay. In addition rapid phage inactivation by joint fluid and cells might have contributed towards drop in their titer. Hence, higher phage titers released soon after implantation may be required to tackle the initial bacterial population.

Previous researchers have shown *in vivo* efficacy of different antibiotic coatings on metallic implants in treating bone and joint infections [43, 91–94]. Lucke et al.[94] demonstrated that gentamicin coating of metallic implants significantly reduced implant-related osteomyelitis in rats. On the contrary, a previous study in a rabbit intramedullary screw *S*. *aureus* osteomyelitis model, found that minocycline and rifampin sprayed onto implant was only partially effective in preventing colonization of implant and infection of the bone [93]. However, in the present study phage and linezolid coated implants were able to significantly reduce the adhered viable bacteria on the implants and surrounding tissue due to their dual action. Also, there was no emergence of resistant mutants during the entire treatment period in any of the phage and /or linezolid implanted mice.

The additive effect seen with dual agents (phage and linezolid) has also been reported by past researchers. They have reported phage-antibiotic synergism (PAS) with both bacteriostatic as well as bactericidal antibiotics [95–100]. Comeau et al. [101] reported that antibiotics, finally block bacterial cell division and this altered physiological state permits faster assembly of phages that leads to faster cell lysis. Similar observation was seen in an earlier in vitro study in which sub-lethal concentrations of linezolid and tetracycline enhanced phage MR-5 plaque size as well as increased its adsorption rate, decreased latent period and enhanced the burst size [98]. It was observed that antibiotics such as linezolid (protein synthesis inhibitors) halt the bacterial growth thus, making the conditions more conducive for phage assembly, lysis and higher progeny production, leading to enhanced overall lytic effect of phage MR-5 [98]. This explains for the additive effect observed in this study that led to maximum reduction of bacterial burden and fastest resolution of experimental implant infection seen in mice with dual coated K-wire.

The combination therapy in turn led to improved locomotion and motor-function/ balancing act of mice and significantly decreased the infection associated inflammation. This is supported by earlier observation that both phages as well as linezolid exhibit significant immunomodulatory potential [102–105]. Letkiewicz and colleagues [106] found strong experimental evidence to show that purified phages decreased activation of the inflammatory cytokine nuclear factor-κB, reduced T-cell adhesion and also inhibited formation of reactive oxygen species by neutrophils. Pabary et al.[107] recently reported that bacteriophages reduced both bacterial load and inflammation in a murine model of *P*. *aeruginosa* lung infection. According to these workers this may be useful in treating cystic fibrosis patients. Recently, Matsumoto and co-workers [108] showed that linezolid exhibited significant anti-inflammatory activity in carrageenan induced rat paw oedema model whereas vancomycin, teicoplanin, arbekacin, and daptomycin showed no such effect. This suggests the protective effects of linezolid and other newer oxazolidinone class of antibiotics for use in chronic inflammatory conditions. Late or chronic stage prosthetic infections are complicated with an acutely inflamed joint, poor mobility, consistent pain that may be associated with systemic features of sepsis[109,110]. Therefore the combination of phage and linezolid is definitely an attractive option showing not only maximal decrease in bacterial load but also maximum decrease in associated inflammation.

In conclusion, the present study reinforces the use of combination coatings of phage and antibiotics as an attractive treatment strategy against orthopaedic implant infections. Although previous researchers have reported advantages of combination therapy given systemically in treatment of prosthetic joint infections [111,112], but use of combination therapy as local delivery has not been looked into. Data also advocates the future use of linezolid and other drugs of the same class (such as tedezolid, ranbezolid) as potential local delivery agents to prevent MRSA mediated implant infections. However, the effect of high doses of these drugs on osteoblast activity needs to be looked into. Since systemic therapy is administered to patients of arthroplasty before, during and after surgery, the effect of combining local delivery of phage with systemically administered antibiotics on the outcome of experimentally induced MRSA implant infection in mice is an additional study required.

This study has clearly highlighted the correlation between functional healing parameters and inflammatory markers in all the respective test groups. This calls for incorporating functional healing tests (i.e locomotion, pain, rotarod test) as important readout parameters in evaluating treatment efficacy and disease progression in Bernthal model of implant infection.

## Materials and Methods

### Bacterial strains and phage used

*Staphylococcus aureus* 43300(MRSA) from ATCC, Manassas, VA was used. The organism was stored in 60% glycerol at -80°C and when necessary, maintained on nutrient agar slants at 4°C.*S*.*aureus* specific bacteriophage, MR-5, which had been isolated and characterized previously in our laboratory was used in the present study [98].

#### Orthopaedic Implants used

Commercially available orthopaedic grade Kirschner-wires (K-wires) of stainless steel (diameter 0.6 mm) were procured from the local market and cut into 20 mm length, cleaned and autoclaved. Three different coating formulations using Hydroxypropylmethylcellulose (HPMC) (K4MP grade; 4000 cps) as the biopolymer (4%w/v) were prepared. These were i) Phage (10^9^ PFU/ml) mixed with HPMC gel, denoted as **H-P** ii) Linezolid (5%w/w) mixed with HPMC gel denoted as **H-L** and iii) Phage as well as Linezolid mixed with HPMC gel, denoted as **H-P-L**. The implants were tested for their adhesive strength, phage stability, phage and linezolid elution kinetics from coated wires and *in vitro* bacterial adherence as described earlier [30].

### Animals

BALB/c female mice, 4–6 weeks old weighing 20–25 gm were used in this study. The animals were obtained from Central Animal House, Panjab University, Chandigarh, India. The animals were kept inpolycarbonate cage, housed in well aerated rooms with a 12-h light/12-h dark cycle at 25 ± 2°C, fed with standard rodent diet and water ad libitum.

### Ethical Statement

The experimental protocols were approved by the Institutional Animal Ethics Committee (Approval ID: IAEC/156) of the Panjab University, Chandigarh, India and performed in accordance with the guidelines of Committee for the Purpose of Control and Supervision of Experiments on Animals (CPCSEA), Government of India, on animal experimentation. All efforts were made to minimize the suffering of animals.

### Preparation of *S*.*aureus* for inoculation into joint space

*S*.*aureus* 43300 was cultivated overnight at 37°C in brain heart infusion broth. Next day, cells were pelleted and washed twice with phosphate-buffered saline (PBS). Bacterial suspension prepared in PBS was adjusted so as to achieve a cell density corresponding to different bacterial inoculums (10^5^,10^6^,10^7^ and 10^8^ CFU/ml). The number of CFU/ml was confirmed by quantitative plate count by spreading each of inoculum on nutrient agar plates.

### Mouse surgical procedure

The mice were anaesthesized by giving *i*.*p* injection of 100mg/kg ketamine and 10 mg/kg xylazine. The surgical procedure was performed according to method of Bernthal et al [43]. After removing the hair from the thigh area, skin of the femur was thoroughly cleaned using 70% alcohol. A skin incision was made over the right knee. The distal right femur was accessed through a medial parapatellar arthrotomy with lateral displacement of quadriceps-patellar complex. After locating the femoral intercondylar notch, the femoral intramedullary canal was manually reamed with a 26 gauge needle and sterile Kirschner (K)-wire (0.6 mm) was surgically placed in a retrograde fashion, protruding into the joint space. The inoculum was injected into the joint space from the cut end of the implant and wound was closed by suturing the area. Buprenorphine (0.1 mg/kg) was administered subcutaneously twice at an interval of 12 hours as ananalgesic to surgically operated animals. Animals were monitored every hour till they fully recovered from the effect of anaesthesia followed by twice daily during the rest of the experimental period. No intraoperative complications were reported and all mice recovered from anaesthesia uneventfully.

### Establishment of *S*.*aureus* 43300 induced joint infection model in BALB/c mice

Bacterial suspension prepared in PBS was adjusted so as to achieve a cell density corresponding to a range of bacterial inoculums (10^5^,10^6^,10^7^ and 10^8^ CFU/ml). Animals were divided into five groups (N = 5) with n = 14 per group. All the animals were surgically operated for placing the K-wire into the femur bone (as described above) followed by bacterial inoculation. Each of the four groups received different inoculum doses. Animals of the fifth group were injected with same volume of PBS (10μl) into the joint space before suturing the wounds. Two animals from each group were sacrificed by cervical dislocation on day 1, 3, 5, 7, 10,15 and 20 post bacterial challenge. The skin of the femur was disinfected with 70% alcohol in order to prevent contamination from skin flora and carefully defleshed. The inserted implant was carefully removed using sterile forcep and dipped in 1 ml PBS. After giving three washings with PBS to the implant, the inserted K-wire was dipped in 1 ml of PBS containing trypsin solution (1%) and sonicated for 15 min to remove all the adhering bacteria. Following sonication, each of the serially diluted trypsin treated preparation was plated on nutrient agar plates for the quantification of viable organisms. Also, the surrounding joint tissue was removed, tissue homogenized, diluted and plated to determine the bacterial burden.

## Efficacy Studies

**Fig 8:**
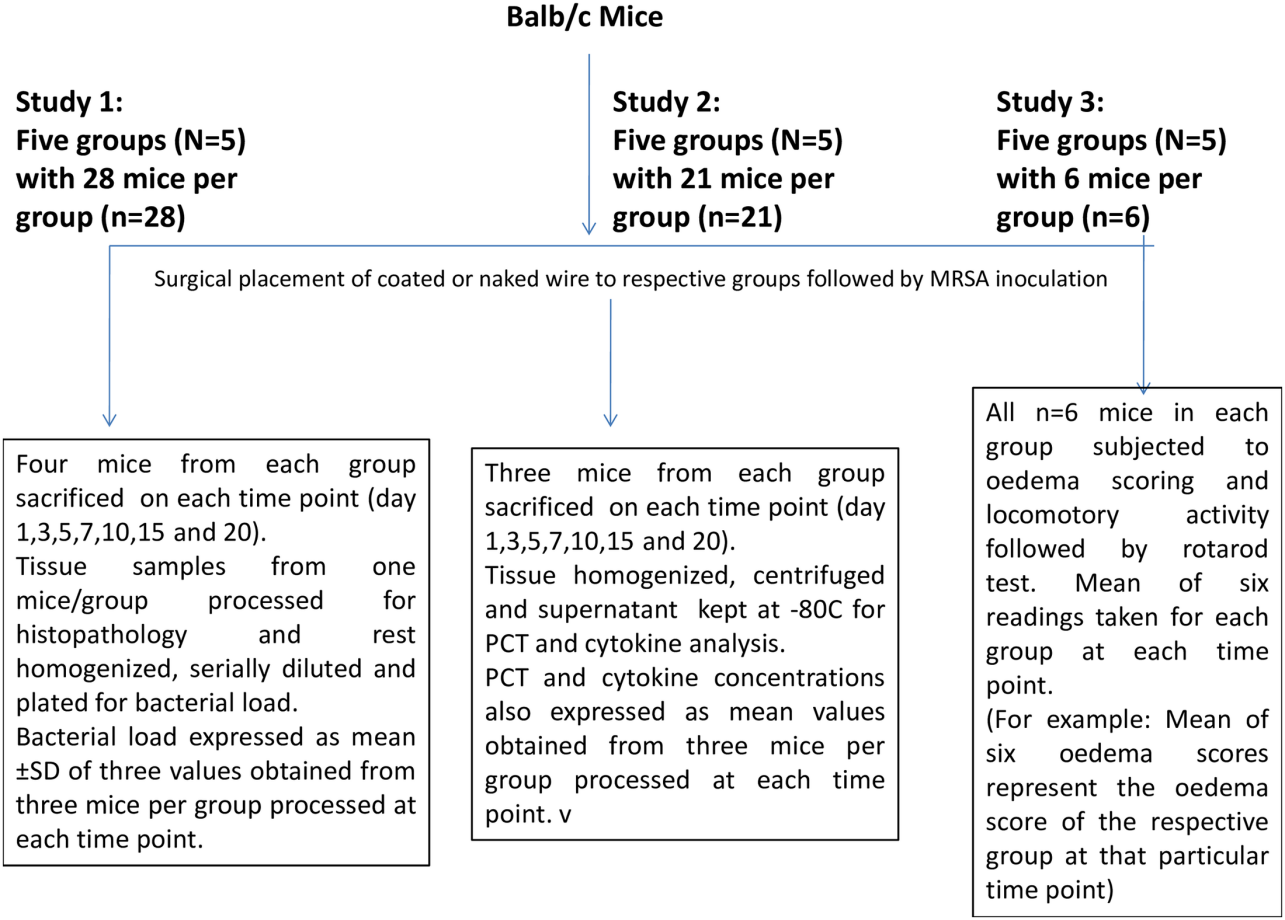
Schematic diagram of the different parameters evaluated to study the course of efficacy.

Therapeutic efficacy of polymer coated K-wires (H-P, H-L, H-P-L) in resolving experimental implant infection of joint in BALB/c mice was studied. The study was divided into three sub-studies as shown in the scheme (Fig 8). All the studies had the same group allocation as discussed below:

BALB/c mice were randomly divided into five groups as follows:

**Group 1:** Sterile naked wire was inserted into the femur followed by infection with *S*.*aureus*43300 (10μl, 10^6^ CFU/ml)

**Group 2:** HPMC (polymer only) coated K-wire was inserted into the femur followed by infection with *S*.*aureus*43300 (10μl, 10^6^CFU/ml)

**Group 3:** Phage coated K-wire (**H-P**) was inserted into the femur followed by followed by infection with *S*.*aureus* 43300.

**Group 4:** Linezolid coated K-wire (**H-L**) was inserted into the femur followed by infection with *S*.*aureus* 43300.

**Group 5:** Dual coated (Phage and linezolid) K-wire (**H-P-L**) was inserted into the femur followed by infection with *S*.*aureus* 43300.

[Note: After surgical placement of K-wires in the femur of mice, all animals were subjected to X-ray examination for confirming the correct placement of the wire (implant). Different parameters were studied following confirmation]

### Oedema Scoring

Oedema of the knee joint area of all test animals was checked with the help of vernier calliper. The normal thickness of the non-affected joint of left knee of the same animal was taken as control. Scoring was done on a scale of 0 to 3. Thickness (in mm) in the range of 3.25–4.04 mm was given a score of 0 i.e no oedema; thickness of 4.10–4.4 was given a score of 1 (mild oedema); thickness of 4.5–4.9 mm was given a score of 2 (moderate oedema) and thickness of >5.0 mm was scored as 3 (severe oedema).

### Functional Healing Parameters

#### Assessment of Locomotor Activity

Locomotor activity was measured as described by Sachdeva et al.[113] by a computerized acto-photometer (IMCORP, India) for 5 min. Mice were individually placed in a transparent plastic cage (30 × 23 × 22 cm^3^) and were allowed to acclimatize to the observation chamber for a period of 2 min. After this, the locomotor activity was noted for 5 minutes (expressed in terms of counts/5 min). One ambulation was recorded when the animal moved from one segment to another. Similarly, one rearing score was recorded when animal stood on its hind limbs with or without support of the wall. Six animals per representative group were subjected to this test. Same number of age matched healthy BALB/c mice (referred to as “control” mice) without any surgery were also tested.

#### Assessment of motor function and joint strength

The rotarod performance test is based on the ability of rodent to stay on the rotating rod. This is done to measure the balancing potential of the animal. This test was performed according to the method of Sachdeva et al [113]. Mice were subjected to motor function evaluation by placing them individually on rota rod, which was adjusted to the speed of 25 rpm. The fall-off time was recorded for each mouse and the longest period any animal stayed on the rod was 300 sec. Six animals per representative group were subjected to this test. Same number of age matched healthy BALB/c mice (referred to as “control” mice) without any surgery were also tested.

### Bacterial burden and phage titer

The number of adhered bacteria on the inserted implant and in the surrounding joint tissue was determined on different days post-infection. Three animals (n = 3) from each group were killed on day 1, 3, 5, 7, 10, 15 and 20 post bacterial challenge. The skin of the femur was disinfected with 70% alcohol in order to prevent contamination from skin flora. The area was carefully defleshed, inserted wire was removed using sterile forcep and dipped in 1 ml PBS. After giving three washings with PBS to the implant, the inserted K-wire was dipped in 1 ml of PBS containing trypsin solution (1%) and sonicated for 15 min to remove adhering bacteria. Following sonication, each of the serially diluted trypsin treated preparation was plated on nutrient agar plates for the quantification of viable organisms. Also, the surrounding joint tissue and femur bone were carefully removed. The bone was cut into small segments and tissue and bone sample suspended in PBS (pH 7.2) followed by homogenization using manual glass homogenizer. The homogenate was diluted and plated to determine the bacterial burden. The same homogenate was subjected to centrifugation (15,000 rpm for 20 min at 4°C) followed by filtration of the supernatant through 0.22 μm pore-size filter prior to conducting phage titration.

### Histopathological Analysis

Extent of injury caused by *S*.*aureus* and healing of infected joint tissue in different groups was assessed on the basis of histopathological examination of injured and recovered paw according to the method of Brans et al [114]. The sections were picked on separate slides, stained with hematoxylin and eosin (Hi-Media, Mumbai) and examined under a microscope to evaluate the extent of damage.

### Pro-Calcitonin (PCT) and Cytokine levels

PCT is a more specific marker of bacterial inflammation as its level does not rise in response to viral infection or non-bacterial inflammation. Hence, mouse PCT levels in the tissue homogenate on different days post infection was determined using commercially available kit (Bio-Medical Assay, Immunoconcept, India).

Since inflammatory cytokines are thought to be good markers for the severity of bacterial infections, the levels of pro-inflammatory (IL-1β, TNF-α) and anti-inflammatory (IL-10)was measured in the tissue homogenate of mice on different days post infection by using commercial enzyme-linked immunosorbent assay kits (BD Biosciences, Pharmingen, CA, USA).

### Screening of resistant mutants

Bacteria detached from implants from H-P, H-L and H-P-L in mice by day 7 were screened for the emergence of resistance to phage and linezolid. Bacteria detached from implanted K-wire were washed twice and cell density was adjusted to 10^8^ CFU/ml. This was then spread plated on Mueller–Hinton agar plates supplemented with linezolid (8 mg/L). In addition, bacteria detached from H-L and H-P-L implants (adjusted to 10^5^ CFU/ml) were subjected to broth dilution method to determine MIC as recommended by Clinical and Laboratory Standards Institute (CLSI) [115].

For studying the sensitivity of bacteria to phage, bacteria detached from H-P and H-P-L implants were used in plaque assay with phage added at MOI-1 and 10.

### Statistical analysis

All data are expressed as mean ± standard deviation of replicated values where indicated. The statistical significance of differences between groups was determined by the Student’s t-test(two groups), one-way ANOVA followed by a Tukey test using Sigma Stat, Graph pad prism(Graph pad software, San Diego, CA). p value of less than 0.05 was considered significant whereas p value of less than 0.01was considered highly significant.

## Supporting Information

S1 Fig Percentage mortality in mice implanted with naked K-wire receiving different bacterial inoculums to respective groups (n = 21). (TIFF)

S1 File Establishment of *S*.*aureus* mediated murine model of joint infection. (DOCX)

S1 Table Bacterial counts (Log CFU/ml) in joint tissue of mice (implanted with naked wire) post infection. NA: Not available for bacterial load estimation due to 100% mortality. Each data point represents mean ± S.D of three values. Error bars represent S.D.
(DOCX)
