# Supplementary figures and images for: In Vivo Assessment of Phage and Linezolid Based Implant Coatings for Treatment of Methicillin Resistant S. aureus (MRSA) Mediated Orthopaedic Device Related Infections
Source: PLoS One. 2016 Jun 22;11(6):e0157626. doi: 10.1371/journal.pone.0157626 (PMC4917197; doi:10.1371/journal.pone.0157626)

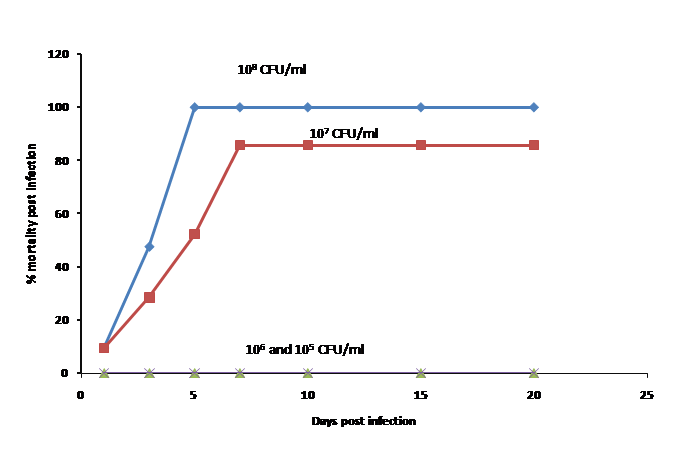

Supplement: S1 Fig — (TIFF) [file pone.0157626.s001.tiff]
