# Supplementary material for: In Vivo Assessment of Phage and Linezolid Based Implant Coatings for Treatment of Methicillin Resistant S. aureus (MRSA) Mediated Orthopaedic Device Related Infections
Source: PLoS One. 2016 Jun 22;11(6):e0157626. doi: 10.1371/journal.pone.0157626 (PMC4917197; doi:10.1371/journal.pone.0157626)
Supplement: S1 File — (DOCX) [file pone.0157626.s002.docx]

**S1 File : Establishment of *S.aureus* mediated murine model of joint infection**

**S1 Table:** Bacterial counts (Log CFU/ml) in joint tissue of mice (implanted with naked wire ) post infection

| **Days** | **10^8^ CFU/ml** | **10^7^ CFU/ml** | **10^6^ CFU/ml** | **10^5^ CFU/ml** |
| --- | --- | --- | --- | --- |
| **1** | 8.11±0.12 | 7.56±0.14 | 6.87±0.10 | 5.41±0.11 |
| **3** | 8.97±0.11 | 8.18±0.11 | 7.61±0.11 | 5.98±0.13 |
| **5** | NA | 8.97±0.08 | 8.11±0.09 | 6.01±0.09 |
| **7** | NA | NA | 6.9±0.12 | 4.41±0.10 |
| **10** | NA | NA | 5.14±0.07 | 3.44±0.12 |
| **15** | NA | NA | 2.76±0.13 | - |
| **20** | NA | NA | - | - |

**NA:** Not available for bacterial load estimation due to 100% mortality. Each data point represents mean ± S.D of three values. Error bars represent S.D.

Results from S1-Table and S1 Fig. indicate that at bacterial inoculums dose of 10^8^ CFU/ml, initial mortality of 10% was observed on day 1 post-infection that gradually increased to 100% by day 5.Similarly, at 10^7^ CFU/ml , mortality of 52.3% was observed by day 5 which increased to 85.7% thereafter. The surviving animals (n=3) showed complete necrosis with shrinking of the infected limb and hence were not processed for load determination. A lower dose of 10^6^ CFU/ml however, showed a consistent development of an acute infection of the joint tissue that progressed through a two week time period with no mortality observed in test mice. It enabled to track the disease progression in mice implanted with naked wire and allowed direct comparisons of all parameters in treated groups vs untreated mice.

**S1 Fig:** **Percentage mortality in mice implanted with naked K-wire receiving different bacterial inoculums to respective groups (n=21).**
