# Supplementary material for: In Vivo Assessment of Phage and Linezolid Based Implant Coatings for Treatment of Methicillin Resistant S. aureus (MRSA) Mediated Orthopaedic Device Related Infections
Source: PLoS One. 2016 Jun 22;11(6):e0157626. doi: 10.1371/journal.pone.0157626 (PMC4917197; doi:10.1371/journal.pone.0157626)
Supplement: S1 Table — NA: Not available for bacterial load estimation due to 100% mortality. Each data point represents mean ± S.D of three values. Error bars represent S.D. (DOCX) [file pone.0157626.s003.docx]

**S1 Table:** Bacterial counts (Log CFU/ml) in joint tissue of mice (implanted with naked wire ) post infection

| **Days** | **10^8^ CFU/ml** | **10^7^ CFU/ml** | **10^6^ CFU/ml** | **10^5^ CFU/ml** |
| --- | --- | --- | --- | --- |
| **1** | 8.11±0.12 | 7.56±0.14 | 6.87±0.10 | 5.41±0.11 |
| **3** | 8.97±0.11 | 8.18±0.11 | 7.61±0.11 | 5.98±0.13 |
| **5** | NA | 8.97±0.08 | 8.11±0.09 | 6.01±0.09 |
| **7** | NA | NA | 6.9±0.12 | 4.41±0.10 |
| **10** | NA | NA | 5.14±0.07 | 3.44±0.12 |
| **15** | NA | NA | 2.76±0.13 | - |
| **20** | NA | NA | - | - |

**NA:** Not available for bacterial load estimation due to 100% mortality. Each data point represents mean ± S.D of three values. Error bars represent S.D.
